# Association of Open Reduction and Internal Fixation With Volar Locking Plate for Distal Radius Fractures With Patient-Reported Outcomes in Older Adults: A Network Meta-analysis

**DOI:** 10.1001/jamanetworkopen.2023.18715

**Published:** 2023-06-16

**Authors:** Mayank Jayaram, Shannon M. Wood, Robert L. Kane, Lan-Yan Yang, Kevin C. Chung

**Affiliations:** 1 Medical Student, University of Michigan Medical School, Ann Arbor; 2 Master’s Student, Johns Hopkins Bloomberg School of Public Health, Baltimore, Maryland; 3 Division of Plastic Surgery, Department of Surgery, The Medical University of South Carolina, Charleston; 4 Biostatistics Unit, Clinical Trial Center, Chang Gung Memorial Hospital, Taoyuan City, Taiwan; 5 Section of Plastic Surgery, University of Michigan Medical School, Ann Arbor

## Abstract

**Importance:**

Randomized clinical trials (RCTs) and meta-analyses have reported inconsistent conclusions regarding optimal distal radius fracture (DRF) treatment in older adults and are limited due to the inclusion of cohort studies with small sample sizes. A network meta-analysis (NMA) addresses these limitations by only synthesizing direct and indirect evidence from RCTs and may clarify optimal DRF treatment in older adults.

**Objective:**

To examine DRF treatment results in optimal short-term and intermediate-term patient-reported outcomes.

**Data Sources:**

Searches of MEDLINE, Embase, Scopus, and Cochrane Central Register of Controlled Trials were conducted for RCTs that investigated DRF treatment outcomes in older adults between January 1, 2000, and January 1, 2022.

**Study Selection:**

Randomized clinical trials including patients with a mean age of 50 years or older that compared the following DRF treatments were eligible for inclusion: casting, open reduction and internal fixation with volar lock plating (ORIF), external fixation, percutaneous pinning, and nail fixation.

**Data Extraction and Synthesis:**

Two reviewers independently completed all data extraction. An NMA aggregated all direct and indirect evidence among DRF treatments. Treatments were ranked by surface under the cumulative ranking curve score. Data are reported as standard mean differences (SMDs) and 95% CIs.

**Main Outcomes and Measures:**

The primary outcome was short-term (≤3 months) and intermediate-term (>3 months to 1 year) Disabilities of the Arm, Shoulder and Hand (DASH) questionnaire scores. Secondary outcomes included Patient-Rated Wrist Evaluation (PRWE) scores and 1-year complication rates.

**Results:**

In this NMA, 23 RCTs consisting of 3054 participants (2495 women [81.7%]) with a mean (SD) age of 66 (7.8) years were included. At 3 months, DASH scores were significantly lower for nail fixation (SMD, −18.28; 95% CI, −29.93 to −6.63) and ORIF (SMD, −9.28; 95% CI, −13.90 to −4.66) compared with casting. The PRWE scores were also significantly lower for ORIF (SMD, −9.55; 95% CI, −15.31 to −3.79) at 3 months. In the intermediate term, ORIF was associated with lowered DASH (SMD, −3.35; 95% CI, −5.90 to −0.80) and PRWE (SMD, −2.90; 95% CI, −4.86 to −0.94) scores. One-year complication rates were comparable among all treatments.

**Conclusions and Relevance:**

The findings of this NMA suggest that ORIF may be associated with clinically significant improvements in short-term recovery compared with casting for multiple patient-reported outcomes measures with no increase in 1-year complication rates. Shared decision-making with patients may be useful to identify patient preferences regarding recovery to determine optimal treatment.

## Key Points

**Question:**

What are the treatment outcomes for distal radius fractures in older adults in the short-term (≤3 months) and intermediate-term (>3 months to 1 year) postoperative periods?

**Findings:**

In this systematic review and network meta-analysis of 23 randomized clinical trials including 3054 participants, distal radius fracture treatment with open reduction and internal fixation with volar locking plate was associated with clinically and statistically significant improvements in short-term patient-reported outcomes assessed with the Disabilities of the Hand and Shoulder Questionnaire and Patient-Rated Wrist Evaluation.

**Meaning:**

The findings of this study suggest that open reduction and internal fixation with volar locking plate may achieve greater patient-reported quality of life in the early postoperative recovery period compared with casting alone.

## Teaser

This meta-analysis examines short- and intermediate-term outcomes among older adults with optimal distal radius fracture treated with casting, open reduction and internal fixation with volar lock plating, external fixation, percutaneous pinning, and nail fixation.

## Introduction

Nearly 50% of all distal radius fractures (DRFs) occur in adults older than 65 years, accounting for 20% of fractures in this patient population.^1,2^ Additionally, the economic burden of DRFs in older adults is estimated to reach $600 million in the next 3 years, a 20% increase since 2005.^3^ The American Academy of Orthopedic Surgeons has published treatment guidelines for DRFs that recommend casting for adults older than 65 years, citing evidence that 1-year outcomes are similar regardless of treatment received.^4^ However, a randomized clinical trial (RCT) demonstrated that surgical treatment with open reduction and internal fixation with volar lock plating (ORIF) results in better short-term outcomes (≤3 months) compared with casting.^5^ Older adults have reported that maintaining autonomy during recovery and returning to daily activities as quickly as possible are crucial to the recovery process, so the earlier return to function offered by surgery is a priority for many patients.^6,7^ Therefore, treating all older adults with casting based on chronologic age may adversely affect quality of life for those who would have preferred an earlier return to activities of daily living.

Several meta-analyses have been performed to compare operative vs nonoperative treatment of DRFs.^8,9,10,11,12,13,14^ However, many of these meta-analyses are limited because they include patients younger than 50 years or include more retrospective and prospective cohort studies than RCTs. Moreover, these studies typically evaluate long-term outcomes (≥1 year) and group all surgical treatments into 1 category rather than separating by treatment type. There are substantial differences in DRF outcomes among various surgical treatments, and grouping all of them into 1 category does not provide sufficient evidence to identify the optimal DRF treatment in older adults.^10,15^ The limitations of traditional meta-analyses can be overcome by performing a network meta-analysis (NMA). An NMA uses direct comparisons from multiple RCTs to make indirect comparisons between other treatment options. For example, if one study compares ORIF with casting and another study compares casting with external fixation, an NMA can make an indirect comparison between external fixation and ORIF by using casting as a constant. Therefore, NMAs can include combinations of RCTs that were not previously used in traditional meta-analyses, thereby capturing the full breadth of DRF outcomes studies in the literature and producing level 1A evidence (systematic review of randomized clinical trials) to estimate which treatment modality achieves superior patient-reported outcomes (PROs) in the early recovery period.

Previous NMAs on DRF outcomes have evaluated long-term outcomes (≥1-year follow-up), whereas others did not present PROs and focused on patients younger than 50 years.^16,17,18^ These NMAs were not designed to detect differences in outcomes across treatment types in older adults during the short and intermediate time frames, when differences in recovery are most relevant to patients. Therefore, we conducted an NMA to investigate the short-term (≤3 months) and intermediate-term (>3 months to 1 year) outcomes of RCTs across multiple surgical options and casting. The purpose of this NMA was to collate data from all DRF RCTs published between January 1, 2000, and January 1, 2022, in older adults to compare the particular treatment modalities in the early recovery period. We hypothesized that patients undergoing surgery who receive ORIF may have the largest improvements in PRO scores in the short-term and intermediate-term recovery periods compared with other treatment modalities in older adults.

## Methods

### Selection Criteria

In this network meta-analysis, we searched MEDLINE (PubMed), Embase (Elsevier), Scopus (Elsevier), and Cochrane Central Register of Controlled Trials for peer-reviewed RCTs evaluating surgical and casting treatments for DRFs in older adults from January 1, 2000, to January 31, 2022. A research librarian assisted in the development of the search strategy and algorithm provided in eTable 1 in Supplement 1. Surgical treatments of interest included ORIF, external fixation, percutaneous pinning, and nail fixation. Studies that had a mean participant age of 50 years and older were included in this analysis. eTable 2 in Supplement 1 provides a list of the inclusion and exclusion criteria. This NMA is registered with the National Institutes of Health PROSPERO research database and followed the Cochrane Confidence in Network Meta-analysis tool. This NMA also followed the Preferred Reporting Items for Systematic Reviews and Meta-analyses (PRISMA) reporting guideline.^19,20^

### Data Extraction and Risk of Bias

All articles were screened by reviewers (M.J., S.M.W.) trained in systematic reviews and meta-analyses. The same 2 reviewers independently examined the title and abstract for articles obtained from the initial search. After review of the titles and abstracts, full-text articles were obtained and uploaded to the web-based systematic review software Rayyan for an in-depth evaluation.^21^ Article characteristics, patient demographic characteristics, follow-up periods, and outcomes data were collected. Discrepancies were discussed by the 2 reviewers until a consensus was reached. If a disagreement persisted, a third reviewer (R.L.K.) was available. Each article underwent a risk-of-bias assessment according to the Cochrane Collaboration guidelines (eTable 3 in Supplement 1). The reviewers categorized the risk of bias in each article as low, high, or unknown across 6 domains: selection, performance, detection, attrition, reporting, and other potential bias.

### Statistical Analysis

Outcome measurements for the analysis were determined after data collection. Disabilities of the Arm, Shoulder and Hand (DASH) and Patient-Rated Wrist Evaluation (PRWE) questionnaires were the most frequently reported outcome measurements in the literature. Studies that did not include DASH and PRWE scores were not included in the analysis. Patients use DASH and PRWE questionnaires, scaled from 0 (no disability) to 100 (fully impaired), to rate their functionality. The data were split into short-term outcomes (≤3 months) and intermediate-term outcomes (>3 months to 1 year), consistent with a previous meta-analysis.^9^ Secondary outcomes included Patient-Rated Wrist Evaluation (PRWE) scores and 1-year complication rates.

For the analysis, a series of pairwise meta-analyses were conducted to measure the direct comparison between treatments. A nodal map was created to establish the geometry among the treatments. Next, an NMA was used to examine outcomes by combining direct and indirect evidence across all treatments. A random-effects model was used for the analysis to account for any heterogeneity that may be present among the study methods. For all analyses, we calculated and report the standard mean differences (SMDs) with the corresponding 95% CIs. A surface under the cumulative ranking curve (SUCRA) value was used to determine the ranking of treatments. SUCRA is measured on a scale of 0 to 1. The closer the SUCRA value is to 1, the higher the likelihood that it is the best or one of the best treatment options, whereas a lower SUCRA score indicates a higher likelihood that the treatment is ranked lower than other treatments.^22^ Because SUCRA analysis identifies the probability that a certain DRF treatment is the optimal treatment, it is a helpful supplement for interpreting pairwise comparisons between treatments by minimizing the effect of small sample sizes on treatment ranking.

## Results

**Figure 1.**
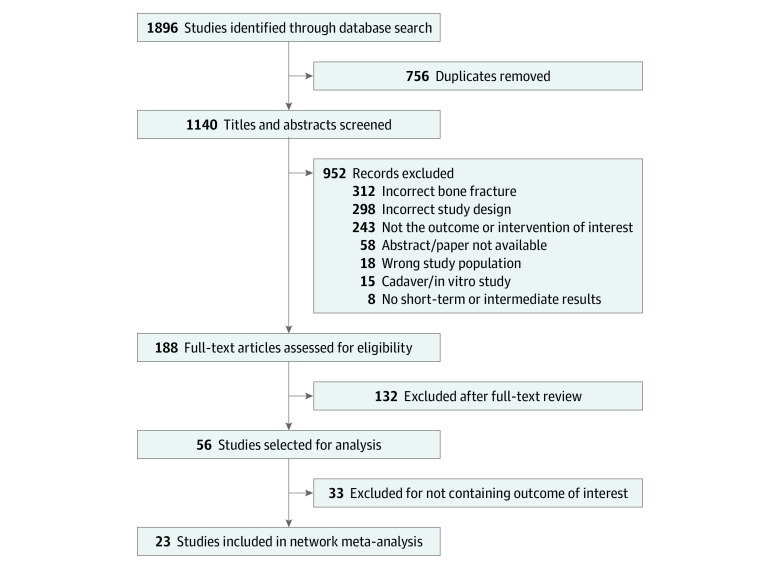
Flowchart of Study Selection

The search strategy yielded 1140 studies; 188 articles were eligible for full-text review. Fifty-six articles originally met all inclusion criteria for the analysis, but only 41% of these articles shared an outcome measure. Therefore, 23 articles were included in the final analysis (Figure 1).^23,24,25,26,27,28,29,30,31,32,33,34,35,36,37,38,39,40,41,42,43,44,45^ Thirteen studies assessed short-term patient-reported outcomes with DASH and PRWE; intermediate-term outcomes were assessed in 18 studies with DASH and 15 studies with PRWE. Of the included studies, 20 evaluated ORIF, 11 evaluated casting, 6 evaluated external fixation, 5 evaluated percutaneous pinning, and 4 studies evaluated nail fixation. A total of 3054 study participants (2495 women [81.7%]; 559 men [18.3%]) were included in the analysis. The mean sample size was 68 (range, 31-500) participants per study. Participants’ age ranged from 19 to 90 years (median, 64.3; IQR, 59.6-71.6 years) with a mean (SD) age of 66 (7.8) years. Fifty-seven percent of the studies used the AO (Arbeitsgemeinschaft für Osteosynthesefragen) Classification system^46^ to report fracture type. Of the studies that reported fracture classification, type A fractures were the most common (930), followed by type C (819) and type B (20). Table 1 includes a list of patient characteristics. Race and ethnicity information was not included because the articles included in the analysis did not collect these data.

**Table 1.** Patient Characteristics

Characteristic	No. (%)
Total participants	3054
Age, y	
Mean (SD)	66 (7.8)
Range	19-90
Median (IQR)	64.3 (59.6-71.6)
Sex	
Female	2495 (81.7)
Male	559 (18.3)
Dominant hand^a^	908 (46.6)
AO classification^a^	1769 (57.4)
Type A	930 (52.6)
Type B	20 (1.1)
Type C	819 (46.3)
Median follow-up (IQR), wk	52 (26)
Missing/lost to follow-up	325 (10.6)

Abbreviation: AO, Arbeitsgemeinschaft für Osteosynthesefragen.

^a^ Not all studies reported this information and percentages were determined from the studies that reported these data.

A risk of bias assessment was performed, and a moderate level of bias was present (eTable 3 in Supplement 1). Nearly all studies were unable to blind participants and health care professionals given the nature of the treatment methods, but approximately 65% of the studies specified using an independent assessor for measuring outcomes. It is unclear whether an independent assessor was used in approximately 35% of the included studies; however, the results of a sensitivity analysis performed with those studies excluded were comparable to the primary analysis (eFigure in Supplement 1). Complications were compared across treatment modalities and were found to be similar among all treatments (eTable 4 in Supplement 1). Of the studies that reported complication rates, external fixation had the highest average complication rate of 0.35 per participant followed by casting (0.30), and percutaneous pinning had the lowest average complication rate (0.17) per participant.

### Network Map

**Figure 2.**
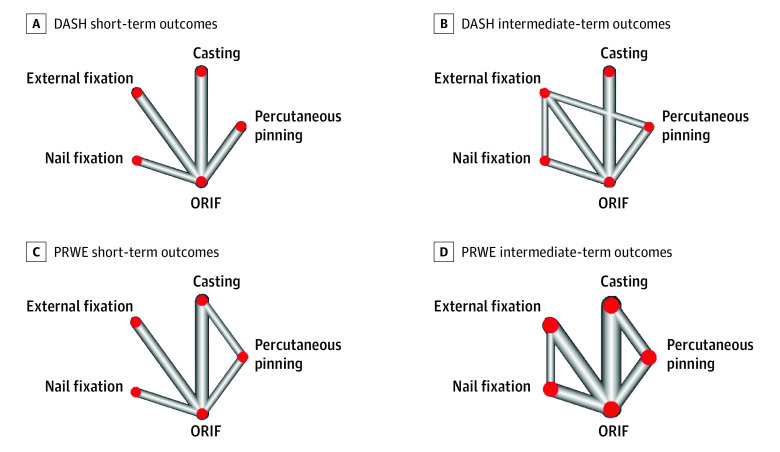
Nodal Network Map for Disabilities of the Arm, Shoulder and Hand (DASH) Questionnaire and Patient-Rated Wrist Evaluation (PRWE) Outcomes DASH short-term (A) and intermediate-term (B) outcomes and PRWE short-term (C) and intermediate-term (D) outcomes. ORIF indicates open reduction and internal fixation with volar lock plating.

A network nodal map was created for each outcome (Figure 2). The width of the lines represents the number of direct comparisons between treatments, and the size of the nodes represents the proportion of patients who received that treatment. A moderate level of heterogeneity (DASH: 43.2%, PRWE: 40.8%) was present within the intermediate-term results (>3 months to 1 year), whereas a high degree of heterogeneity (DASH: 61.7%, PRWE: 85.1%) was present within the short-term results (≤3 months). In the 23 included studies, a total of 59 pairwise comparisons were performed across the short-term and intermediate-term results for each outcome (eFigure in Supplement 1).

**Figure 3.**
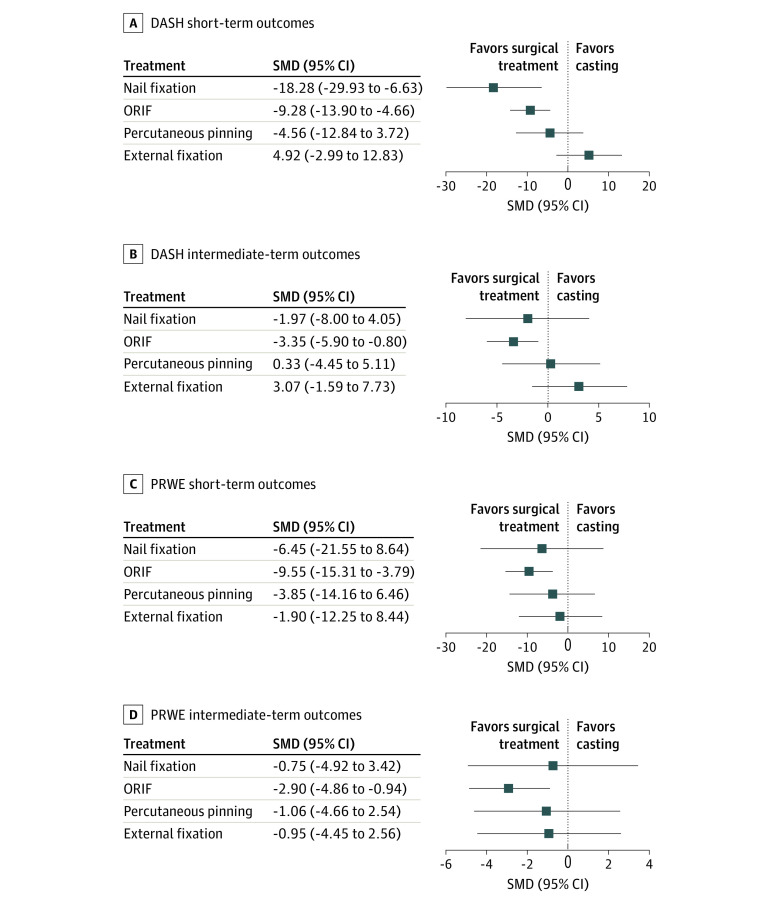
Outcomes for Disabilities of the Arm, Shoulder and Hand (DASH) Questionnaire and Patient-Rated Wrist Evaluation (PRWE) DASH short-term (A) and intermediate-term (B) outcomes and PRWE short-term (C) and intermediate-term (D) outcomes. ORIF indicates open reduction and internal fixation with volar lock plating; SMD, standard mean difference.

For DASH short-term results, there was a significant reduction in DASH scores for nail fixation (SMD, −18.28; 95% CI, −29.93 to −6.63) and ORIF (SMD, −9.28; 95% CI, −13.90 to −4.66) compared with casting (Figure 3A). After 3 months, only ORIF (SMD, −3.55; 95% CI, −5.90 to −0.80) showed significantly lower DASH scores compared with casting (Figure 3B). Similarly, for PRWE short-term and intermediate-term outcomes, ORIF (short term: SMD, −9.55; 95% CI, −15.31 to −3.79; intermediate term: SMD, −2.90; 95% CI, −4.86 to −0.94) showed significantly lower PRWE scores (Figure 3C and D). Next, a network estimate or a weighted average of direct and indirect evidence was calculated to make pairwise comparisons across all treatments (eFigure in Supplement 1). External fixation (SMD, 6.42; 95% CI, 2.52-10.32) was associated with an increase in DASH scores compared with ORIF between 3 months and 1 year. The network estimate further supports the use of ORIF over casting to decrease PRWE scores (short term: SMD, −9.55; 95% CI, −15.31 to −3.79; intermediate term: SMD, −2.90; 95% CI, −4.8 to −0.94).

### Ranking of Treatments

The SUCRA values for each treatment were calculated for the DASH and PRWE questionnaires (Table 2). For DASH short-term results, nail fixation (0.99) was identified as the best treatment, followed by ORIF (0.74). For the intermediate-term results, ORIF (0.91) was identified as the best treatment, followed by nail fixation (0.70). For PRWE outcomes, ORIF (short term: 0.84, intermediate term: 0.85) was identified as the best treatment, followed by percutaneous pinning (short-term: 0.70, intermediate term: 0.61).

**Table 2.** SUCRA Values for Treatments

Treatment	SUCRA
**DASH**	
Short term (≤3 mo)	
Nail fixation	0.99
ORIF	0.74
Percutaneous pinning	0.49
Casting	0.25
External fixation	0.04
Intermediate term (>3 mo to 1 y)	
ORIF	0.91
Nail fixation	0.70
Casting	0.44
Percutaneous pinning	0.35
External fixation	0.10
**PRWE**	
Short term (≤3 mo)	
ORIF	0.84
Percutaneous pinning	0.70
External fixation	0.45
Nail fixation	0.27
Casting	0.24
Intermediate term (>3 mo to 1 y)	
ORIF	0.85
Percutaneous pinning	0.61
External fixation	0.42
Nail fixation	0.33
Casting	0.29

Abbreviations: DASH, Disabilities of the Arm, Shoulder and Hand questionnaire; ORIF, open reduction and internal fixation with volar lock plating; PRWE, Patient-Rated Wrist Evaluation; SUCRA, surface under the cumulative ranking curve.

## Discussion

The results of our NMA and systematic review show level 1A evidence that ORIF is associated with better short-term PROs in older adults compared with other DRF treatment modalities with no increase in 1-year complication rates. To our knowledge, this is the first NMA conducted of studies of older adults (age ≥50 years) that compared DASH and PRWE scores among various DRF treatments in the early and intermediate recovery periods. Patient-reported outcomes such as DASH and PRWE provide comprehensive insights into recovery after treatment by including both functional elements and psychosocial components related to recovery. By using PROs as our primary outcome, we identified that casting in all patients with DRFs does not align with optimal patient-perceived recovery compared with other treatment modalities. Therefore, we would recommend offering surgical treatment of DRFs to older adults with excellent physiologic health, regardless of chronologic age, while engaging in a shared decision-making process with each patient to tailor optimal management.

After consolidating all direct and indirect comparisons among DRF treatments from 23 RCTs, we observed that older adults receiving ORIF performed 9.28 points better on DASH outcomes and 9.55 points better on the PRWE outcomes by the 3-month recovery period. These values fall within the range of the minimal clinically important difference for DRFs, which is estimated to be between 6.8 and 10.8 for DASH and 8.5 and 11.5 for PRWE outcomes.^47,48^ However, by the intermediate term, the improvements were limited to 3.55 in DASH and 2.90 in PRWE scores after ORIF. Although these values were statistically significant, they did not meet the minimal clinically important difference threshold and were not clinically significant. These findings are consistent with the current literature that suggests long-term recovery is similar regardless of treatment choice. Another notable finding is that nail fixation noted an approximate 18-point improvement in DASH scores in the early recovery period. However, only 1 study included in this analysis investigated nail fixation in the short-term recovery period compared with the 7 ORIF studies included for this time point. Further studies are needed to examine whether nail fixation can provide better outcomes than ORIF in older adults in the short term.

Based on these findings, using chronologic age as a proxy for functional demand and recommending casting for all older adults does not apply equally to all patients. Patients who desire earlier recovery could substantially benefit from ORIF treatment and should be counseled on the advantages of surgical treatment during their clinical visits. Moreover, future DRF studies should focus on identifying which older adults would most greatly benefit from ORIF treatment compared with casting. Stratifying older adults by activity status, level of frailty, or number of comorbidities could be one method to estimate functional demand and integrate physiologic health into treatment decisions.^49,50,51,52^ We advise clinicians to prioritize identifying patient preferences for treatment recovery to assist them in making an informed decision about their treatment plan. Moreover, the consolidation of data from these RCTs suggests that ORIF is the optimal surgical fixation strategy in older adults compared with other methods, such as external fixation and percutaneous pinning. Therefore, older adults who desire surgical fixation and are amenable to volar lock plating should be informed that ORIF is associated with the best short-term outcomes after DRF after consolidating data from multiple RCTs.

An incidental finding from this study was the profound heterogeneity in outcome instruments used and variables reported in the literature. After full-text review, 56 articles had met all inclusion criteria; however, only 41% of those original studies were eligible for inclusion after limiting the included articles to shared outcome measures. Consequently, numerous RCTs could not be studied in this analysis, including the Wrist and Radius Injury Surgical Trial (WRIST),^5^ which was one of the largest multisite RCTs performed in older adults with DRFs. Despite not being able to include all RCTs, the results of this NMA are consistent with findings from WRIST demonstrating higher short-term PRO scores for individuals receiving ORIF compared with casting. Several of the common PROs reported in the literature included instruments such as the DASH, PRWE, Michigan Hand Outcomes Questionnaire, Short Form-36, and visual analog scale. Additionally, functional metrics, such as grip strength, also greatly varied in the literature, sometimes reported in kilograms or other times as a percentage of the uninjured hand. Given the various outcome measurements, a standardized reporting measure for DRF outcome studies would be necessary so that these data can be synthesized appropriately. This could be one opportunity to minimize variation among outcome measures in the literature and provide more comprehensive treatment recommendations for this population.

### Limitations

This study has several limitations. To perform an NMA, we must assume the property of transitivity, which assumes study populations from RCTs are homogeneous enough to make indirect comparisons among different treatment modalities.^51^ Additionally, a major assumption for the property of transitivity is that the distribution of effect modifiers across treatments does not vary significantly across studies. We attempted to ensure the study populations were similar by imposing strict criteria (mean age ≥50 years and examining specific treatments for DRFs), but there could be other differences between the study populations and study designs investigated that were not homogeneous. Additionally, PRO metrics were standardized to improve the homogeneity of the study population. Moreover, the differences between direct and indirect pairwise comparisons were minimal among studies with overlap across the 95% CI. Next, our study did not discriminate based on type of DRF, primary or secondary displacement, or high-energy vs low-energy fracture. Some high-energy fractures from a motor vehicle collision could not safely be treated with casting and would require operative management. Therefore, results could have been different depending on fracture type. A random-effects model was used to minimize potential heterogeneity from these variables. In addition, because casting and ORIF are considered the predominant DRF treatments in older adults, there were few studies that directly compared these treatments with other modalities. As a result, the nodal maps did not close completely and there were fewer pairwise comparisons at certain follow-up points.

## Conclusions

In this NMA, level 1A evidence suggests that ORIF is associated with earlier recovery after DRF treatment in older adults. It may be useful for clinicians to work with patients to determine their preferences for DRF treatment and consider offering ORIF to patients who desire more rapid recovery rather than waiting 1 year for optimal treatment outcomes. Future studies should investigate alternative methods for identifying which patients would benefit most from ORIF treatment in the short term and intermediate term.

## Notes

Supplement 1. **eTable 1.** Search Strategy **eTable 2.** Inclusion and Exclusion Criteria **eFigure.** Network Estimates for (I) DASH and (II) PRWE Short and Intermediate Outcomes **eTable 3.** Traffic Light Plot **eTable 4.** Reported Complications Across Included Studies
eReferences

Supplement 2.
Data Sharing Statement
