# Supplementary material for: Association of Open Reduction and Internal Fixation With Volar Locking Plate for Distal Radius Fractures With Patient-Reported Outcomes in Older Adults: A Network Meta-analysis
Source: JAMA Netw Open. 2023 Jun 16;6(6):e2318715. doi: 10.1001/jamanetworkopen.2023.18715 (PMC10276304; doi:10.1001/jamanetworkopen.2023.18715)
Supplement: Supplement 1. — eTable 1. Search Strategy eTable 2. Inclusion and Exclusion Criteria eFigure. Network Estimates for (I) DASH and (II) PRWE Short and Intermediate Outcomes eTable 3. Traffic Light Plot eTable 4. Reported Complications Across Included Studies eReferences [file jamanetwopen-e2318715-s001.pdf]

## Supplementary Online Content

Jayaram M, Wood SM, Kane RL, Yang LY, Chung KC. Association of open reduction and internal fixation with volar locking plate for distal radius fractures with patient-reported outcomes in older adults: a network meta-analysis. *JAMA Netw Open*. 2023;6(6):e2318715. doi:10.1001/jamanetworkopen.2023.18715

**eTable 1.** Search Strategy

**eTable 2.** Inclusion and Exclusion Criteria

**eFigure.** Network Estimates for (I) DASH and (II) PRWE Short and Intermediate Outcomes

**eTable 3.** Traffic Light Plot

**eTable 4.** Reported Complications Across Included Studies

**eReferences**

This supplementary material has been provided by the authors to give readers additional information about their work.

© 2023 Jayaram M et al. *JAMA Network Open.*

**eTable 1. Search Strategy**

| Database | Search Strategy |
|---|---|
| MEDLINE | ((distal[tiab] OR wrist[tiab] OR wrists[tiab] OR wrist injuries[mh]) AND (radius fractures[mh] OR fracture[tiab] OR fractures[tiab]) OR Smith fracture[tiab] OR smith fractures[tiab] OR Colles fracture[tiab] OR colles fractures[tiab] OR Barton fracture[tiab] OR barton fractures[tiab]) AND<br><br>(surgical procedures, operative[mh] OR fixation[tiab] OR orthopaedic[tiab] OR orthopaedic[tiab] OR orthopedics[tiab] OR orthopedic[tiab]) AND (conservative[tiab] OR non-operative[tiab] OR non-surgical[tiab] OR cast[tiab] OR percutaneous pinning[tiab] OR volar plating[tiab] OR ORIF[tiab] OR "open reduction"[tiab] OR closed reduction[tiab] OR plate[tiab]) AND<br><br>(RCT OR random* OR trial) AND<br><br>(aged[mh] OR older adult [tiab] OR middle aged[mh] OR geriatric[tiab] OR elderly[tiab] OR elder[tiab] OR elders[tiab] OR osteoporo*[tiab]) |
| Embase | ('distal radius fracture'/exp OR ((distal OR wrist* OR wrists OR radius OR radial) NEAR/3 fracture*):ti,ab OR ‘Smith fracture‘:ti,ab OR ‘smith fractures‘:ti,ab OR ‘Colles fracture’:ti,ab OR ‘colles fractures’:ti,ab OR ‘Barton fracture’:ti,ab OR ‘barton fractures’:ti,ab) AND<br><br>('closed reduction (procedure)'/exp OR 'fracture fixation'/exp OR 'fracture treatment'/exp OR 'orthopedic surgery'/exp OR 'closed reduction':ti,ab OR ((‘fixation’:ti,ab OR orthopaedic*:ti,ab OR orthopedics*:ti,ab) AND (‘conservative’:ti,ab OR ‘non-operative’:ti,ab OR ‘non-surgical’:ti,ab OR ‘cast’:ti,ab OR ‘percutaneous pinning’:ti,ab OR ‘volar plating’:ti,ab OR ‘ORIF’:ti,ab OR ‘open reduction’:ti,ab OR ‘plate’:ti,ab))) AND (‘RCT’:ti,ab OR ‘random*’:ti,ab OR ‘trial’:ti,ab) AND ('frail elderly'/exp OR 'very elderly'/exp OR ‘older adult ‘:ti,ab OR 'middle aged'/exp OR ‘geriatric’:ti,ab OR ‘elderly’:ti,ab OR ‘elder’:ti,ab OR ‘elders’:ti,ab OR osteoporo*:ti,ab) |
| Central | (distal NEAR/2 radi* NEAR/2 Fracture*) AND (fixation OR ortho* OR conservative* OR 'non-operative' OR 'non-surgical' OR cast OR pinning OR volar OR plating OR ORIF OR 'open reduction' OR 'closed reduction' OR plate) |
| SCOPUS | (TITLE-ABS("distal") OR TITLE-ABS("wrist*") OR INDEXTERMS("wrist injuries")) AND (INDEXTERMS("radius fractures") OR TITLE-ABS("fracture*")) OR TITLE-ABS("Smith fracture*") OR TITLE-ABS("Colles fracture") OR TITLE-ABS("colles fractures") OR TITLE-ABS("Barton fracture") OR TITLE-ABS("barton fractures") AND<br><br>(INDEXTERMS("Fixation") OR INDEXTERMS("Fracture Fixation") OR TITLE-ABS("orthopaedic") OR TITLE-ABS("orthopaedic") OR TITLE-ABS("orthopedics") OR TITLE-ABS("orthopedic")) AND (TITLE-ABS("conservative") OR TITLE-ABS("non-operative") OR TITLE-ABS("non-surgical") OR TITLE-ABS("cast") OR TITLE-ABS("percutaneous pinning") OR TITLE-ABS("volar plating") OR TITLE-ABS("ORIF") OR TITLE-ABS("open reduction") OR TITLE-ABS("closed reduction") OR TITLE-ABS("plate")) AND (INDEXTERMS(“RCT”) OR TITLE-ABS(“random*”) OR TITLE-ABS(“trial”)) AND<br><br>(INDEXTERMS("elderly") OR INDEXTERMS ("Aged") OR TITLE-ABS("older adult") OR TITLE-ABS("geriatric") OR TITLE-ABS("elder*") OR TITLE-ABS("osteoporo*")) |

© 2023 Jayaram M et al. *JAMA Network Open.*

**eTable 2: Inclusion and Exclusion Criteria**

| Inclusion/Exclusion | Criteria |
|---|---|
| Inclusion | • Randomized Control Trial<br>• Average Adult Age > 50<br>• Distal Radius Fracture<br>• Treatments<br>  o Open Reduction Internal Fixation (Volar Locking Plate System)<br>  o External Fixation<br>  o Percutaneous Pinning<br>  o Nail Fixation<br>  o Casting |
| Exclusion | • Any non-randomized control trials including prospective/retrospective cohort studies, case series/case reports and cross-sectional studies<br>• Average age of adults less than 50 years old<br>• Surgical treatments that are not listed above |

© 2023 Jayaram M et al. *JAMA Network Open.*

**eFigure: Network Estimates for (I) DASH and (II) PRWE short and intermediate outcomes**

I. DASH intermediate outcomes*

a. > 3 Months to 1 Year

| Comparison | Number of Studies | Direct Evidence | I2 | SMD | 95%-CI |
|---|---|---|---|---|---|
| **External Fixation vs Nail Fixation** | | | | | |
| Direct estimate | 1 | 0.36 | | -1.60 | [-11.62; 8.42] |
| Indirect estimate | | | | 8.82 | [ 1.26; 16.37] |
| Network estimate | | | | 5.04 | [ -0.99; 11.08] |
| **External Fixation vs ORIF** | | | | | |
| Direct estimate | 3 | 0.72 | 75% | 6.44 | [ 1.83; 11.04] |
| Indirect estimate | | | | 6.37 | [ -0.96; 13.70] |
| Network estimate | | | | 6.42 | [ 2.52; 10.32] |
| **External Fixation vs Percutaneous Pinning** | | | | | |
| Direct estimate | 1 | 0.37 | | 7.10 | [ -1.07; 15.27] |
| Indirect estimate | | | | 0.22 | [ -6.00; 6.44] |
| Network estimate | | | | 2.74 | [ -2.21; 7.69] |
| **Nail Fixation vs ORIF** | | | | | |
| Direct estimate | 2 | 0.75 | 0% | -1.27 | [ -7.60; 5.05] |
| Indirect estimate | | | | 9.14 | [ -1.70; 19.98] |
| Network estimate | | | | 1.37 | [ -4.09; 6.83] |
| **ORIF vs Percutaneous Pinning** | | | | | |
| Direct estimate | 3 | 0.81 | 66% | -5.00 | [ -9.50; -0.50] |
| Indirect estimate | | | | 1.88 | [ -7.35; 11.12] |
| Network estimate | | | | -3.67 | [ -7.72; 0.37] |

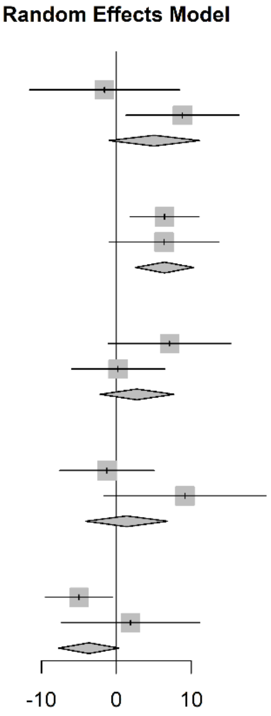


© 2023 Jayaram M et al. *JAMA Network Open.*

I. PRWE (a) short-term outcomes and (b) intermediate outcomes

a. ≤ 3 Months

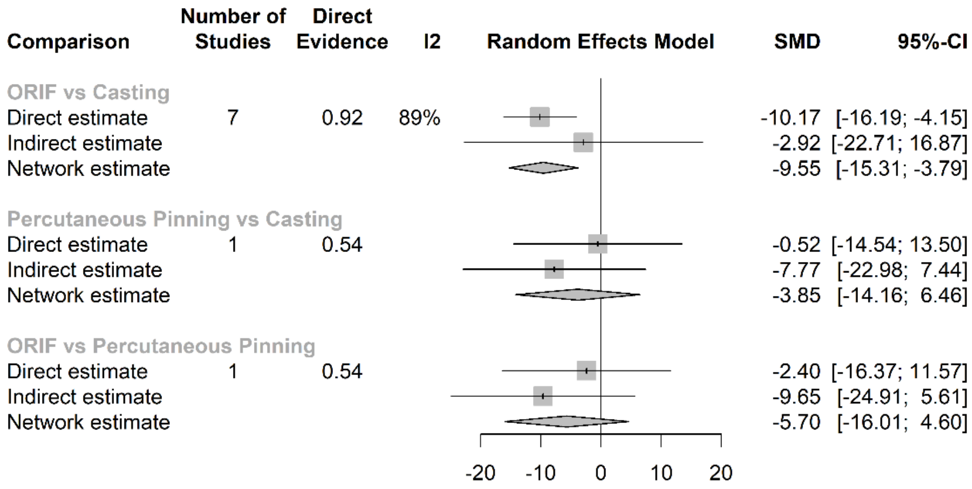


© 2023 Jayaram M et al. *JAMA Network Open.*

b. > 3 Months to 1 Year

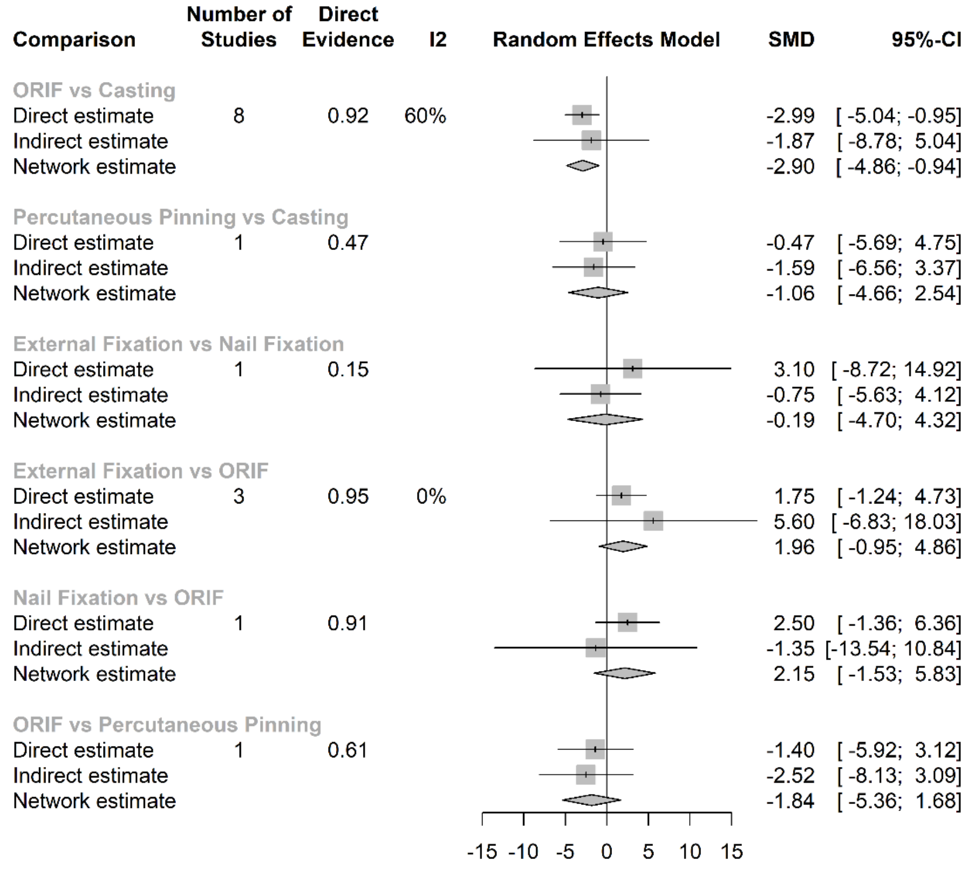


© 2023 Jayaram M et al. *JAMA Network Open.*

| | Arora 2011 | Bartl 2014 | Belloti 2010 | Chappius 2010 | Costa 2014 | Costa 2022 | Eikrem 2021 | Hassellund 2021 | Hollevoet 2011 | Larouche 2016 | Lawson 2021 | Ludvigsen 2020 | Marcheiz 2010 | Mellstrand 2016 | Mulders 2019 | Safi 2013 | Saving 2019 | Schonneman 2011 | Selles 2021 | Sirnio 2019 | Tahir 2020 | Wei 2009 | Wilcke 2011 |
|---|---|---|---|---|---|---|---|---|---|---|---|---|---|---|---|---|---|---|---|---|---|---|---|
| Random Sequence Generation | + | + | + | + | + | + | + | + | + | - | + | + | + | + | + | + | + | + | + | + | + | + | + |
| Allocation Concealment | + | + | + | + | + | + | + | + | - | - | ? | + | + | + | + | + | + | + | + | + | + | + | + |
| Blinding of Participants | - | - | - | ? | - | - | - | - | - | - | - | - | ? | - | - | + | - | - | - | ? | - | - | - |
| Blinding of Personnel | - | - | - | - | - | - | - | - | - | - | - | - | ? | - | - | ? | - | - | - | ? | - | - | - |
| Blinding of Outcome Assessor | + | + | - | - | + | + | + | + | ? | ? | + | + | ? | - | + | ? | - | ? | ? | ? | + | + | ? |
| Incomplete outcome data | + | + | + | + | + | + | + | + | ? | + | + | + | + | + | + | + | + | + | + | + | + | + | + |
| Selective Reporting | + | + | + | + | + | + | + | + | + | + | + | + | + | + | + | + | + | + | + | + | + | + | + |
| Other potential problems increasing risk of bias | + | + | + | + | + | - | - | - | + | + | + | + | - | + | - | + | + | + | - | + | + | + | - |

**eTable 3: Traffic Light Plot[1-23]**

© 2023 Jayaram M et al. *JAMA Network Open.*

**eTable 4. Reported Complications Across Included Studies**

| Treatment Type | Studies | Participants | Total Complications | Average Complications Per Participant | References |
|---|---|---|---|---|---|
| ORIF | 20 | 1,196 | 286 | 0.24 | Arora et al, Bartl et.al, Chappius et al, Costa et al, Eikrem et al, Hassellund et al, Hollevoet et al, Larouche et al*, Lawson et al, , Ludvigsen et al, Marcheix et al, Mellstrand et al, Mulders et.al, Safi et al, Saving et al, Selles et al, Sirnio et al, Tahir et.al, Wei et al, Wilcke et al |
| Casting | 11 | 840 | 252 | 0.30 | Arora et al, Bartl et.al, Costa 2022 et al, Hassellund et al, Larouche et al*, Lawson et al, Mulders et.al, Saving et al, Selles et al, Sirnio et al, Tahir et al |
| External Fixation | 6 | 281 | 97 | 0.35 | Belloti et al, Ludvigsen et al, Mellstrand et al, Schonnemann et al, Wei et al, Wilcke et al |
| Percutaneous Pinning | 5 | 599 | 103 | 0.17 | Belloti et al, Costa et al, Costa 2022 et al, Hollevoet et al, Marcheix et al |
| Nail Fixation | 4 | 138 | 35 | 0.25 | Chappius et al, Eikrem et al, Safi et al, Schonnemann et al |

*Studies that did not report total complications separated by treatment type.

© 2023 Jayaram M et al. *JAMA Network Open.*

**eReferences**

1. Arora R, Lutz M, Deml C, Krappinger D, Haug L, Gabl M. A prospective randomized trial comparing nonoperative treatment with volar locking plate fixation for displaced and unstable distal radial fractures in patients sixty-five years of age and older. *J Bone Joint Surg Am*. 2011;93(23):2146-2153. Medline:22159849 doi:10.2106/JBJS.J.01597

2. Bartl C, Stengel D, Bruckner T, Gebhard F; ORCHID Study Group. The treatment of displaced intra-articular distal radius fractures in elderly patients. *Dtsch Arztebl Int*. 2014;111(46):779-787. Medline:25491556 doi:10.3238/arztebl.2014.0779

3. Belloti JC, Tamaoki MJ, Atallah AN, Albertoni WM, dos Santos JB, Faloppa F. Treatment of reducible unstable fractures of the distal radius in adults: a randomised controlled trial of De Palma percutaneous pinning versus bridging external fixation. *BMC Musculoskelet Disord*. 2010;11:137. Medline:20587064 doi:10.1186/1471-2474-11-137

4. Chappuis J, Bouté P, Putz P. Dorsally displaced extra-articular distal radius fractures fixation: dorsal IM nailing versus volar plating—a randomized controlled trial. *Orthop Traumatol Surg Res*. 2011;97(5):471-478. Medline:21659011 doi:10.1016/j.otsr.2010.11.011

5. Costa ML, Achten J, Parsons NR, et al; DRAFFT Study Group. Percutaneous fixation with Kirschner wires versus volar locking plate fixation in adults with dorsally displaced fracture of distal radius: randomised controlled trial. *BMJ*. 2014;349:g4807. Medline:25096595 doi:10.1136/bmj.g4807

6. Eikrem M, Brannsten H, Bjørkøy D, Lian T, Madsen JE, Figved W. Volar locking plate versus dorsal locking nail-plate fixation for dorsally displaced unstable extra-articular distal radial fractures: functional and radiographic results from a randomized controlled trial. *JB JS Open Access*. 2021;6(4):e21.00068. Medline:34651093 doi:10.2106/JBJS.OA.21.00068

7. Hassellund SS, Williksen JH, Laane MM, et al. Cast immobilization is non-inferior to volar locking plates in relation to QuickDASH after one year in patients aged 65 years and older: a randomized controlled trial of displaced distal radius fractures. Bone Joint J. 2021;103-B(2):247-255. doi:10.1302/0301-620X.103B2.BJJ-2020-0192.R2

8. Hollevoet N, Vanhoutie T, Vanhove W, Verdonk R. Percutaneous K-wire fixation versus palmar plating with locking screws for Colles' fractures. *Acta Orthop Belg*. 2011;77(2):180-187. Medline:21667729

9. Lawson A, Naylor JM, Buchbinder R, et al; Combined Randomised and Observational Study of Surgery for Fractures in the Distal Radius in the Elderly (CROSSFIRE) Study Group. Surgical plating vs closed reduction for fractures in the distal radius in older patients: a randomized clinical trial. *JAMA Surg*. 2021;156(3):229-237. Medline:33439250 doi:10.1001/jamasurg.2020.5672

© 2023 Jayaram M et al. *JAMA Network Open.*

10. Ludvigsen T, Matre K, Gudmundsdottir RS, Krukhaug Y, Dybvik EH, Fevang JM. Surgical treatment of distal radial fractures with external fixation versus volar locking plate: a multicenter randomized controlled trial. *J Bone Joint Surg Am*. 2021;103(5):405-414. Medline:33369985 doi:10.2106/JBJS.20.00275

11. Marcheix PS, Dotzis A, Benkö PE, Siegler J, Arnaud JP, Charissoux JL. Extension fractures of the distal radius in patients older than 50: a prospective randomized study comparing fixation using mixed pins or a palmar fixed-angle plate. *J Hand Surg Eur Vol*. 2010;35(8):646-651. Medline:20237186 doi:10.1177/1753193410364179

12. Mellstrand Navarro C, Ahrengart L, Törnqvist H, Ponzer S. Volar locking plate or external fixation with optional addition of K-wires for dorsally displaced distal radius fractures: a randomized controlled study. *J Orthop Trauma*. 2016;30(4):217-224. Medline:26709818 doi:10.1097/BOT.0000000000000519

13. Mulders MAM, Walenkamp MMJ, van Dieren S, Goslings JC, Schep NWL; VIPER Trial Collaborators. Volar plate fixation versus plaster immobilization in acceptably reduced extra-articular distal radial fractures: a multicenter randomized controlled trial. *J Bone Joint Surg Am*. 2019;101(9):787-796. Medline:31045666 doi:10.2106/JBJS.18.00693

14. Safi A, Hart R, Těknědžjan B, Kozák T. Treatment of extra-articular and simple articular distal radial fractures with intramedullary nail versus volar locking plate. *J Hand Surg Eur Vol*. 2013;38(7):774-779. Medline:23442339 doi:10.1177/1753193413478715

15. Saving J, Severin Wahlgren S, Olsson K, et al. Nonoperative treatment compared with volar locking plate fixation for dorsally displaced distal radial fractures in the elderly: a randomized controlled trial. *J Bone Joint Surg Am*. 2019;101(11):961-969. Medline:31169572 doi:10.2106/JBJS.18.00768

16. Schønnemann JO, Hansen TB, Søballe K. Randomised study of non- bridging external fixation compared with intramedullary fixation of unstable distal radial fractures. *J Plast Surg Hand Surg*. 2011;45(4- 5):232-237. Medline:22150146 doi:10.3109/2000656X.2011.613243

17. Selles CA, Mulders MAM, Winkelhagen J, van Eerten PV, Goslings JC, Schep NWL; VIPAR Collaborators. Volar plate fixation versus cast immobilization in acceptably reduced intra-articular distal radial fractures: a randomized controlled trial. *J Bone Joint Surg Am*. 2021;103(21):1963-1969. Medline:34314402 doi:10.2106/JBJS.20.01344

18. Sirniö K, Leppilahti J, Ohtonen P, Flinkkilä T. Early palmar plate fixation of distal radius fractures may benefit patients aged 50 years or older: a randomized trial comparing 2 different treatment protocols. *Acta Orthop*. 2019;90(2):123-128. Medline:30669897 doi:10.1080/17453674.2018.1561614

19. Tahir M, Khan Zimri F, Ahmed N, et al. Plaster immobilization versus anterior plating for dorsally displaced distal radial fractures in elderly patients in Pakistan. *J Hand Surg Eur Vol*. 2021;46(6):647-653. Medline:33487060 doi:10.1177/1753193420977780

© 2023 Jayaram M et al. *JAMA Network Open.*

20. Wei DH, Raizman NM, Bottino CJ, Jobin CM, Strauch RJ, Rosenwasser MP. Unstable distal radial fractures treated with external fixation, a radial column plate, or a volar plate: a prospective randomized trial. *J Bone Joint Surg Am*. 2009;91(7):1568-1577. Medline:19571078 doi:10.2106/JBJS.H.00722

21. Wilcke MK, Abbaszadegan H, Adolphson PY. Wrist function recovers more rapidly after volar locked plating than after external fixation but the outcomes are similar after 1 year. *Acta Orthop*. 2011;82(1):76-81. Medline:21281262 doi:10.3109/17453674.2011.552781

22. Costa ML, Achten J, Ooms A, et al; DRAFFT2 Collaborators. Surgical fixation with K-wires versus casting in adults with fracture of distal radius: DRAFFT2 multicentre randomised clinical trial. *BMJ*. 2022;376:e068041. Medline:35045969 doi:10.1136/bmj-2021- 068041

23. Larouche J, Pike J, Slobogean GP, et al. Determinants of functional outcome in distal radius fractures in high-functioning patients older than 55 years. *J Orthop Trauma*. 2016;30(8):445-449. Medline:26978132 doi:10.1097/BOT.0000000000000566

© 2023 Jayaram M et al. *JAMA Network Open.*
