# Supplementary material for: Association of Open Reduction and Internal Fixation With Volar Locking Plate for Distal Radius Fractures With Patient-Reported Outcomes in Older Adults: A Network Meta-analysis
Source: JAMA Netw Open. 2023 Jun 16;6(6):e2318715. doi: 10.1001/jamanetworkopen.2023.18715 (PMC10276304; doi:10.1001/jamanetworkopen.2023.18715)
Supplement: Supplement 2. — Data Sharing Statement [file jamanetwopen-e2318715-s002.pdf]

Data Sharing Statement

Jayaram. Association of Open Reduction and Internal Fixation With Volar Locking Plate for Distal Radius Fractures With Patient-Reported Outcomes in Older Adults. *JAMA Netw Open*. Published June 16, 2023. doi:10.1001/jamanetworkopen.2023.18715

Data
Data available: No

Additional Information
Explanation for why data not available: We do not plan to share our data.
